# Resolvin D1 attenuates inflammation in lipopolysaccharide-induced acute lung injury through a process involving the PPARγ/NF-κB pathway

**DOI:** 10.1186/1465-9921-13-110

**Published:** 2012-12-02

**Authors:** Zenglin Liao, Jiajia Dong, Wei Wu, Ting Yang, Tao Wang, Lingli Guo, Lei Chen, Dan Xu, Fuqiang Wen

**Affiliations:** 1 Division of Pulmonary Diseases, State Key Laboratory of Biotherapy of China, and Department of Respiratory Medicine, West China Hospital of Sichuan University, Chengdu, Sichuan, 610041, China

**Keywords:** Acute lung injury, Docosahexaenoic acid, Lipid mediators, NF-κB, Peroxisome proliferator-activated receptor gamma, Resolvin D1

## Abstract

**Background:**

Docosahexaenoic acid (DHA) and DHA-derived lipid mediators have recently been shown to possess anti-inflammatory and pro-resolving properties. In fact, DHA can down-regulate lipolysaccharide (LPS)-induced activation of NF-κB via a PPARγ-dependent pathway. We sought to investigate the effects of the novel DHA-derived mediator resolvin D1 (RvD1) on LPS-induced acute lung injury and to determine whether these effects occur via a PPARγ-dependent pathway.

**Methods:**

BALB/c mice aged 6–8 weeks were randomly divided into seven groups: two control groups receiving saline or RvD1 (600 ng) without LPS; a control group receiving LPS only; an experimental group receiving RvD1 (300 ng) or RvD1 (600 ng), followed by LPS; a group receiving the PPARγ antagonist GW9662; and a group receiving GW9662, then RvD1 (600 ng) and finally LPS. LPS (50 μM) and saline were administered intratracheally. RvD1 was injected intravenously 24 h and 30 min before LPS, while GW9662 was injected intravenously 30 min before RvD1. Mice were killed at 6, 12, and 24 h. Samples of bronchoalveolar lavage fluid (BALF) were analyzed for cell counts and cytokine analysis. Lung tissues were collected for histology, Western blotting and electrophoretic mobility shift assays (EMSAs).

**Results:**

At all three time points, groups receiving either dose of RvD1 followed by LPS had significantly lower total leukocyte counts and levels of TNF-α and IL-6 levels in BALF than did the group given only LPS. RvD1 markedly attenuated LPS-induced lung inflammation at 24 h, based on hematoxylin-eosin staining of histology sections. RvD1 activated PPARγ and suppressed IκBα degradation and NF-κB p65 nuclear translocation, based on Western blots and EMSAs. The PPARγ inhibitor GW9662 partially reversed RvD1-induced suppression of IκBα degradation and p65 nuclear translocation.

**Conclusions:**

These results suggest that RvD1 may attenuate lung inflammation of LPS-induced acute lung injury by suppressing NF-κB activation through a mechanism partly dependent on PPARγ activation.

## Background

Acute lung injury (ALI) and its severe form, acute respiratory distress syndrome (ARDS), are inflammatory disorders of the lung caused by pneumonia, sepsis, trauma and/or aspiration [1]. ALI is characterized by hypoxemia, non-cardiogenic pulmonary edema, low lung compliance and widespread capillary leakage. ALI and ARDS contribute to significant morbidity and mortality in intensive care units. Although improvements in mechanical ventilation and supportive care have increased the survival rates of patients with ALI/ARDS, pharmacological treatments have not improved these rates [2]; as a result, in-hospital mortality rates remain high: 38.5% for ALI and 41.1% for ARDS [3]. Thus, there is a need for innovative pharmacological therapies to improve clinical outcomes.

Inflammation plays an important role in the pathophysiology of ALI. While effective host defense depends on a self-limiting inflammatory response, excessive or uncontrolled inflammation can lead to tissue injury, chronic inflammation, scarring, and fibrosis [4]. Inflammation was previously thought to resolve through a passive process, but recent findings demonstrate that resolution is an active, regulated process of clearing inflammatory exudates [5]. Using a lipidomics approach based on liquid chromatography-tandem mass spectrometry, researchers have identified new lipid mediators of spontaneous resolution [4]. These endogenous lipid mediators are biosynthesized from omega-3 polyunsaturated fatty acids (ω-PUFA) and can accelerate the resolution of acute inflammation and restoration of tissue homeostasis. Resolvin D1 (C_22_H_32_O_5_) is one of these lipid mediators and is derived from the ω-PUFA docosahexaenoic acid (DHA) [4].

Feeding patients with ARDS enterically with supplements enriched with ω-PUFA improves clinical outcomes, including shortening the time on mechanical ventilation and the stay in the intensive care unit [6]. The therapeutic effects of ω-PUFA in inflammatory disease occur via activation of peroxisome proliferator-activated receptor gamma (PPARγ) and inhibition of NF-κB activation [7]. PPARγ is a ligand-activated nuclear transcription factor that plays important roles in cellular differentiation, cancer, inflammation, insulin sensitization, atherosclerosis, and metabolic diseases [8]. When activated, PPARγ binds to the PPAR-response element and represses or induces transcription of target genes [9]. Previous studies have shown ω-PUFA to be natural ligands of PPARγ [10]. PPARγ inhibits NF-κB and thereby suppresses several inflammatory processes [11]. DHA acts through a PPARγ-dependent pathway to down-regulate lipopolysaccharide (LPS)-induced activation of NF-κB in human kidney-2 cells [12].

Since RvD1 derives from DHA and shows anti-inflammatory effects in models of allergic airway response [13] and in patients with peritonitis and renal ischemia–perfusion injury [6], we hypothesized that RvD1 may also promote the resolution of ALI. Since ω-PUFA are natural ligands of PPARγ, we further hypothesized that RvD1 may act through a mechanism dependent on PPARγ. To test these ideas, we used a mouse model of LPS-induced ALI to examine whether RvD1 accelerates the resolution of inflammation and, if so, whether it acts via a PPARγ/NF-κB pathway.

## Material and methods

### Animal groups and treatments

Male BALB/c mice aged 6–8 weeks were purchased from the Experimental Animal Center of Sichuan University. Animals were free of specific pathogens and were kept on a 12 h light/12 h dark cycle at a room temperature of 22 ± 2°C with free access to food and water. Experimental procedures were conducted under aseptic conditions. The study protocol was approved by the Animal Care and Use Committee of West China Hospital.

Mice were randomly divided into seven groups (n = 3 per group): two control groups receiving saline or 600 ng RvD1 without LPS; a control group receiving LPS only; an experimental group receiving 300 ng or 600 ng RvD1, followed by LPS; a group receiving the PPARγ antagonist GW9662; and a group receiving GW9662, then 600 ng RvD1 and finally LPS. The first five groups served to determine the protective effects of RvD1 in LPS-induced ALI. The last two groups, which received the potent PPARγ antagonist GW9662, served to explore whether the effects of RvD1 occur via a PPARγ pathway.

RvD1 (purity ≥ 95%) was purchased from the Cayman Chemical Company (Michigan, USA). Doses of 300 or 600 ng/mouse were chosen based on our own preliminary data and on other studies [14,15]. These doses were administered in 100 μl of sterile saline by tail vein injection 24 h and 30 min before LPS administration; other groups were treated with 100 μl of sterile saline. LPS (*E*. *coli* serotype O111:B4; Sigma-Aldrich, USA) was administered intratracheally during inspiration, at a dose of 50 μg/mouse in 100 μl of sterile saline. Non-LPS groups were treated with 100 μl of sterile saline.

GW9662 (Sigma-Aldrich, USA) was dissolved in 10% DMSO and administered at a dose of 1 mg/kg by tail vein injection 30 min before RvD1 injection; other groups were treated with a suitable volume of 10% DMSO. The dose and timing of GW9662 administration were based on our own preliminary data as well as previous work [16].

### LPS-induced ALI and sample collection

Mice were anesthetized with intraperitoneal 1% sodium pentobarbital (80 mg/kg) and the trachea was exposed using a neck incision. LPS (50 μM) or saline (control) was administered intratracheally. Mice recovered from anesthesia within 1 h. After recovery, mice were returned to their cages and allowed food and water ad libitum. At 6, 12, and 24 h after LPS treatment, mice were sacrificed to allow collection of bronchoalveolar lavage fluid (BALF) and lung tissue. BALF samples were taken from the right lung, after which it was snap-frozen in liquid nitrogen and stored at -80°C for Western blot analysis. The left lung was fixed and embedded in paraffin for morphological and histochemical analyses.

### BALF analysis and cell counts

Mice were exsanguinated via the abdominal aorta, the trachea was cannulated, and the chest cavity was opened via a midline incision. The left main-stem bronchus was ligated, and the right lung was lavaged three times with 0.5 ml of ice-cold sterile saline. In all cases, more than 95% of the total lavage volume (1.5 ml) was recovered. A 0.5-ml aliquot of BALF was used to determine total cell and differential cell counts. The remaining BALF was centrifuged at 1000 *g* for 5 min at 4°C, and the cell-free supernatant was stored at -80°C for analysis of cytokines using enzyme-linked immunosorbent assay (ELISA).

The 0.5-ml aliquot of BALF was subjected to hypotonic shock to lyse red blood cells, then total cell counts were determined using a hemacytometer. Cells were adjusted to a concentration of 5 × 10^5^/ml in supplemented phosphate-buffered saline (PBS). After cytocentrifugation (Cytopro 7620; Wescor, Utah, USA) at 700 rpm for 10 min, cells were stained with Wright's stain. Differential cell counts were made on samples of 200 cells. An experienced investigator blinded to the experimental conditions performed all counts based on standard morphological criteria.

### ELISA for inflammatory cytokines

Concentrations of TNF-α and IL-6 in BALF were determined using commercially available ELISA kits for mouse cytokines (Shanghai ExCell Biology, China) and a Bio-Rad 680 microplate reader with accompanying software (Bio-Rad, Hercules, CA), following the instructions of the manufacturers.

### Evaluation of ALI severity

The left lung was fixed in 4% formaldehyde (pH 7.4), embedded in paraffin, cut into sections 4 mm thick and stained with hematoxylin and eosin (H&E). An ALI score (minimum: 0, maximum: 16) [17] was calculated as an index of the degree of lung injury. The mean score was calculated based on five randomly selected high-power fields (HPF, 400X magnification), and mean scores for different groups were compared.

### Western Blot analysis

Lung tissue samples collected at 6 h after LPS treatment were homogenized, and cytoplasmic and nuclear proteins were extracted separately using the Nuclear and Cytoplasmic Protein Extraction Kit (Viagene Biotech, Ningbo, China) according to the manufacturer's instructions. Nuclear protein extracts were used to detect the NF-κB p65 subunit, PPARγ and histone H3.1; cytoplasmic extracts were used to detect IκBα and β-actin. Mouse polyclonal antibodies were used against IκBα, the NF-κB p65 subunit, and PPARγ (Cell Signaling Technology, USA); histone H3.1 (Signalway Antibody, USA); and β-actin (Santa Cruz Biotechnology, USA). Band intensities for NF-κB p65 subunit and PPARγ were normalized to that of histone H3.1, while the band intensity for IκBα was normalized to that of β-actin. All blotting experiments were performed three times with different mice.

### Electrophoretic mobility shift assay to detect NF-κB

Nuclear protein extracts were analyzed for the presence of NF-κB using a non-radioactive NF-κB electrophoretic mobility shift assay (EMSA) kit (Viagene Biotech Co.). Equal amounts of nuclear protein (5 μg) from each sample were mixed with biotin-labeled oligonucleotide NF-κB probe (5′-AGT TGA GGG GAC TTT CCC AGGC-3′) and analyzed by EMSA following the manufacturer's instructions, as previously described [18]. Each assay was performed three times using extracts from different mice.

### Statistical analysis

All values were expressed as mean ± SD. Differences among multiple groups were analyzed by one-way ANOVA, followed by the Student-Newman-Keuls test. Significance was defined by a p value of 0.05 (two-tailed). All statistical calculations were carried out in SPSS (version 13.0, SPSS Inc., USA).

## Results

### RvD1 reduced the number of leukocytes in BALF

**Figure 1:**
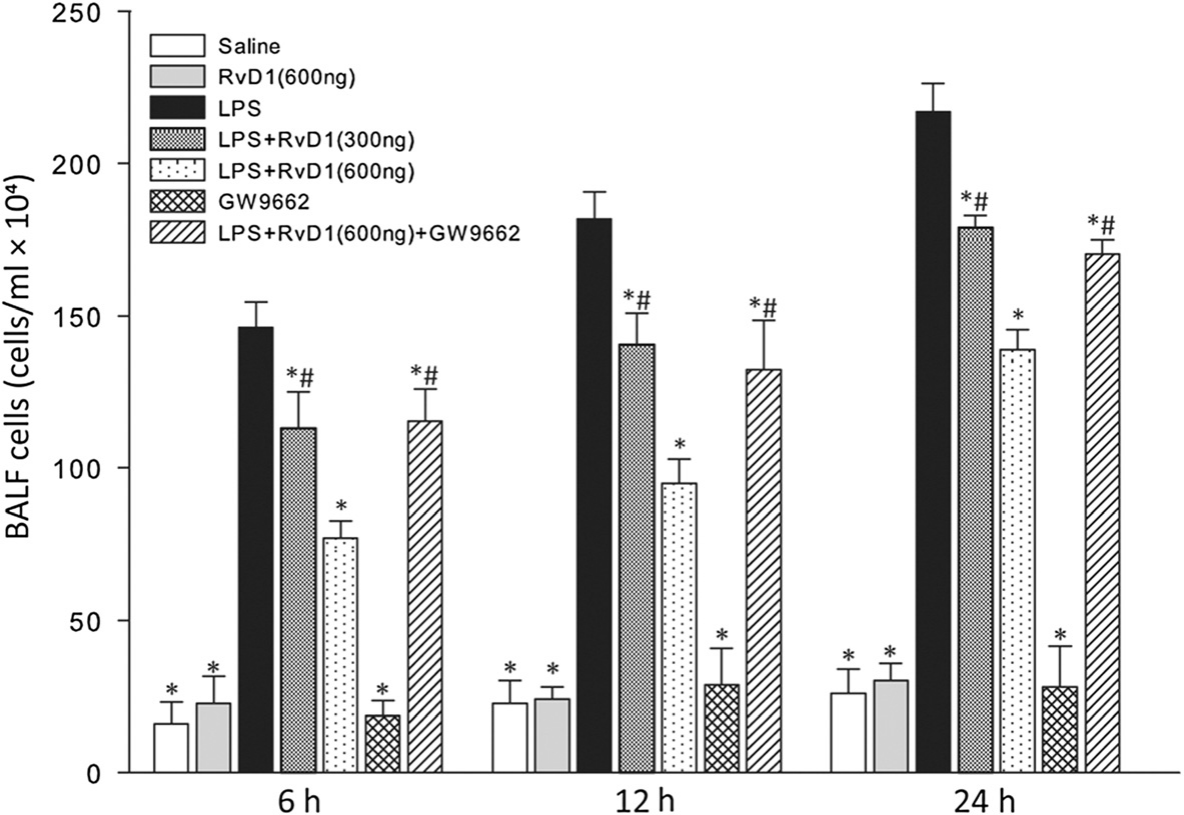
**Effects of RvD1 on total cell counts in BALF from mice subjected to LPS-induced ALI.** BALF samples were collected at 6 h, 12 h, and 24 h after LPS administration. RvD1 reduced the numbers of leukocytes in BALF, and this effect was partially reversed by GW9662. Values are expressed as mean ± SD (n = 3). * p < 0.05 in comparison with the LPS group at each time point. # p < 0.05 in comparison with the LPS + RvD1 (600 ng) group at each time point.

After LPS was administered, total cell counts in BALF increased from 6 h to 24 h, and differential cell counts revealed that the increase in leukocytes was due mainly to neutrophils (Figure 1, Table 1). Numbers of macrophages and lymphocytes, in contrast, did not differ significantly among the groups. At each time point, RvD1 significantly reduced the number of leukocytes, mainly neutrophils, in BALF; this effect was dose-dependent. Administering the PPARγ antagonist GW9662 before injecting 600 ng RvD1 partially reversed the RvD1-induced reduction in leukocyte number.

**Table 1:** **Differential cell counts** (**expressed as cells** × **10**^**4**^) **in BALF from mice subjected to LPS-induced ALI following different treatments**

**Treatment group**	**Time after LPS induction of ALI**								
	**6 h**			**12 h**			**24 h**		
	**Macrophages**	**Neutrophils**	**Lymphocytes**	**Macrophages**	**Neutrophils**	**Lymphocytes**	**Macrophages**	**Neutrophils**	**Lymphocytes**
Saline	15.43 ± 6.80	0.03 ± 0.04	0.55 ± 0.42	22.10 ± 7.10	0.07 ± 0.07	0.50 ± 0.66	25.25 ± 7.99	0.22 ± 0.05	0.53 ± 0.03
RvD1 (600 ng)	21.25 ± 8.89	0.42 ± 0.72	1.00 ± 0.31	22.34 ± 3.70	0.33 ± 0.42	1.33 ± 0.61	28.44 ± 4.29	0.18 ± 0.19	1.71 ± 1.10
LPS	15.07 ± 3.35	128.89 ± 6.45	2.37 ± 0.69	15.83 ± 4.71	162.73 ± 4.00	3.11 ± 1.75	20.74 ± 3.71	193.61 ± 7.65	2.65 ± 1.64
RvD1 (300 ng) + LPS	11.71 ± 3.62	99.42 ± 8.10*#	1.87 ± 2.15	17.41 ± 2.52	121.07 ± 8.46*#	2.19 ± 0.60	20.24 ± 2.78	155.77 ± 5.70*#	2.99 ± 0.46
RvD1 (600 ng) + LPS	10.70 ± 1.23	63.33 ± 2.09*	2.97 ± 2.27	10.39 ± 1.41	81.38 ± 8.00*	3.23 ± 1.38	14.50 ± 1.77	120.59 ± 9.59*	3.91 ± 1.59
GW9662	17.90 ± 4.40	0.21 ± 0.36	0.57 ± 0.34	26.48 ± 10.81	0.48 ± 0.66	1.71 ± 0.98	26.19 ± 12.49	0.30 ± 0.32	1.51 ± 1.22
GW9662 + RvD1 (600 ng) + LPS	13.02 ± 2.61	99.59 ± 7.15*#	2.72 ± 1.43	15.52 ± 0.71	113.52 ± 16.19*#	3.29 ± 0.58	14.15 ± 1.46	152.59 ± 2.91*#	3.60 ± 1.26

Data are mean ± SD, n = 3. BALF, bronchoalveolar lavage fluid. RvD1, resolvin D1. * p < 0.05 in comparison with the LPS group at each time point. # p < 0.05 in comparison with the RvD1 (600 ng) + LPS group at each time point.

### RvD1 reduced concentrations of TNF-α and IL-6 in BALF

**Figure 2:**
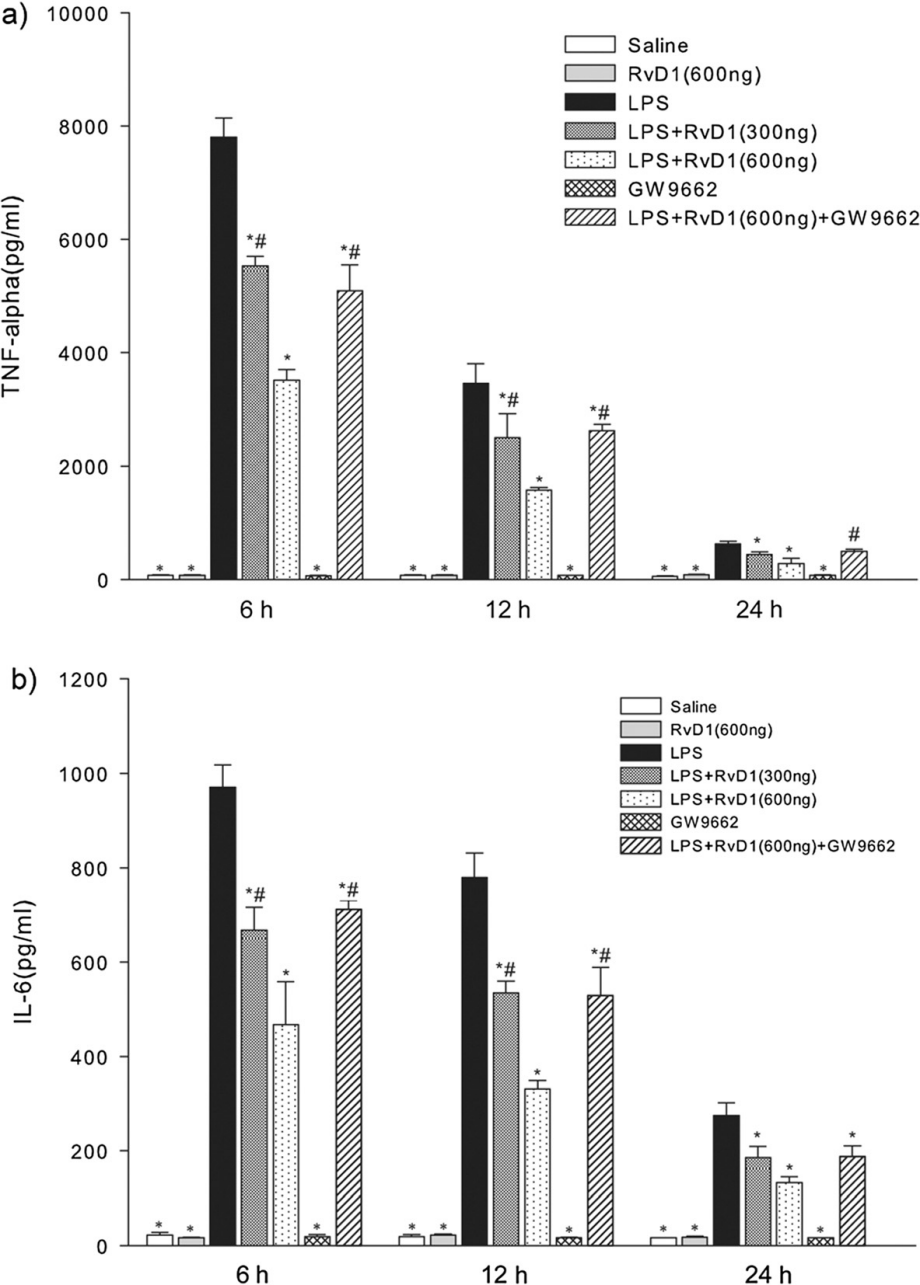
**Effects of RvD1 on the concentrations of (a) TNF-α and (b) IL-6 in BALF of mice subjected to LPS-induced ALI.** BALF samples were collected at 6 h, 12 h, and 24 h after LPS administration. RvD1 reduced the concentrations of TNF-α and IL-6 in BALF, and these effects were partially reversed by GW9662. Values are expressed as mean ± SD (n = 3). * p < 0.05 in comparison with the LPS group at each time point. # p < 0.05 in comparison with the LPS + RvD1 (600 ng) group at each time point.

The levels of both TNF-α and IL-6 in BALF decreased from 6 h to 24 h (Figure 2). At 6 and 12 h, RvD1 down-regulated TNF-α and IL-6 levels in a dose-dependent manner. At 24 h, levels of TNF-α and IL-6 were lower after treatment with 600 ng RvD1 than after treatment with 300 ng, although this difference was not statistically significant. Administering GW9662 before injecting 600 ng RvD1 partially reversed the RvD1-induced down-regulation of TNF-α and IL-6.

### RvD1 attenuated LPS-induced lung inflammation

**Figure 3:**
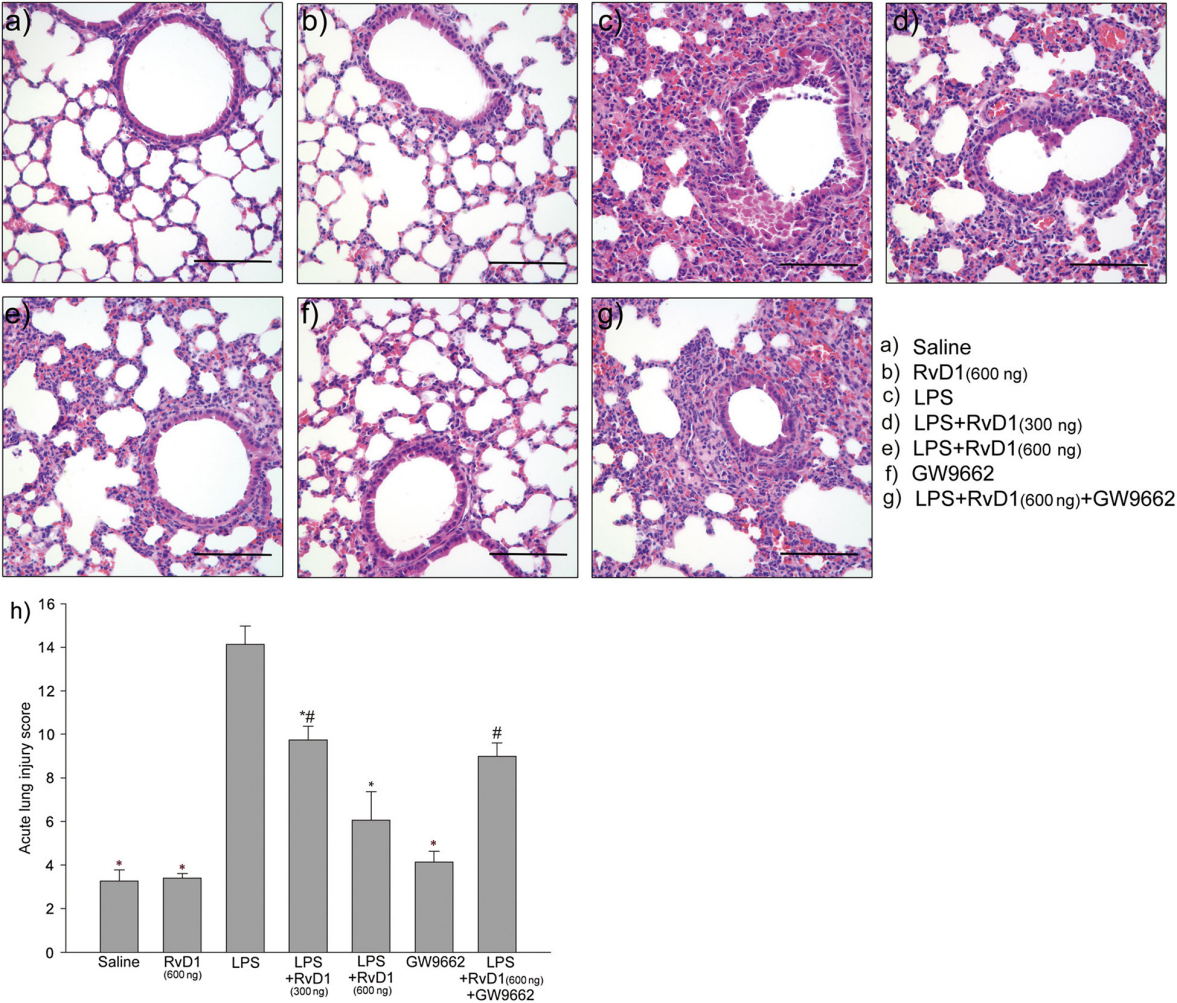
**Effects of RvD1 on histological changes in lung tissues of mice subjected to LPS-induced ALI.** Lung tissue samples were collected at 24 h after LPS administration, and sections were stained with hematoxylin and eosin (H&E). Mice had been treated with the following: **(a)** saline, **(b)** 600 ng RvD1, **(c)** LPS only, **(d)** LPS + RvD1 (300 ng), **(e)** LPS + RvD1 (600 ng), **(f)** GW9662, **(g)** LPS + RvD1 (600 ng) + GW9662. **(h)** The degree of lung injury in tissue sections was assessed based on an ALI score. Values are expressed as mean ± SD (n = 3) .* p < 0.05 in comparison with the LPS group. # p < 0.05 in comparison with the LPS + RvD1 (600 ng) group. Scale bar = 100 μm.

Lung tissue samples were collected 24 h after LPS administration, and sections were stained with H&E to allow determination of an ALI score describing the degree of lung injury. RvD1 attenuated LPS-induced lung inflammation in a dose-dependent manner: mean ALI scores were significantly lower in the group receiving 600 ng RvD1 than in the one receiving 300 ng RvD1 (Figure 3). Administering GW9662 before injecting 600 ng RvD1 prevented this anti-inflammatory effect of RvD1: the ALI score was significantly higher in the group receiving GW9662 followed by 600 ng RvD1 than in the group receiving only 600 ng RvD1.

### Effect of RvD1 on LPS-induced expression of IκBα, NF-κB p-65 subunit and PPARγ in lung tissue

**Figure 4:**
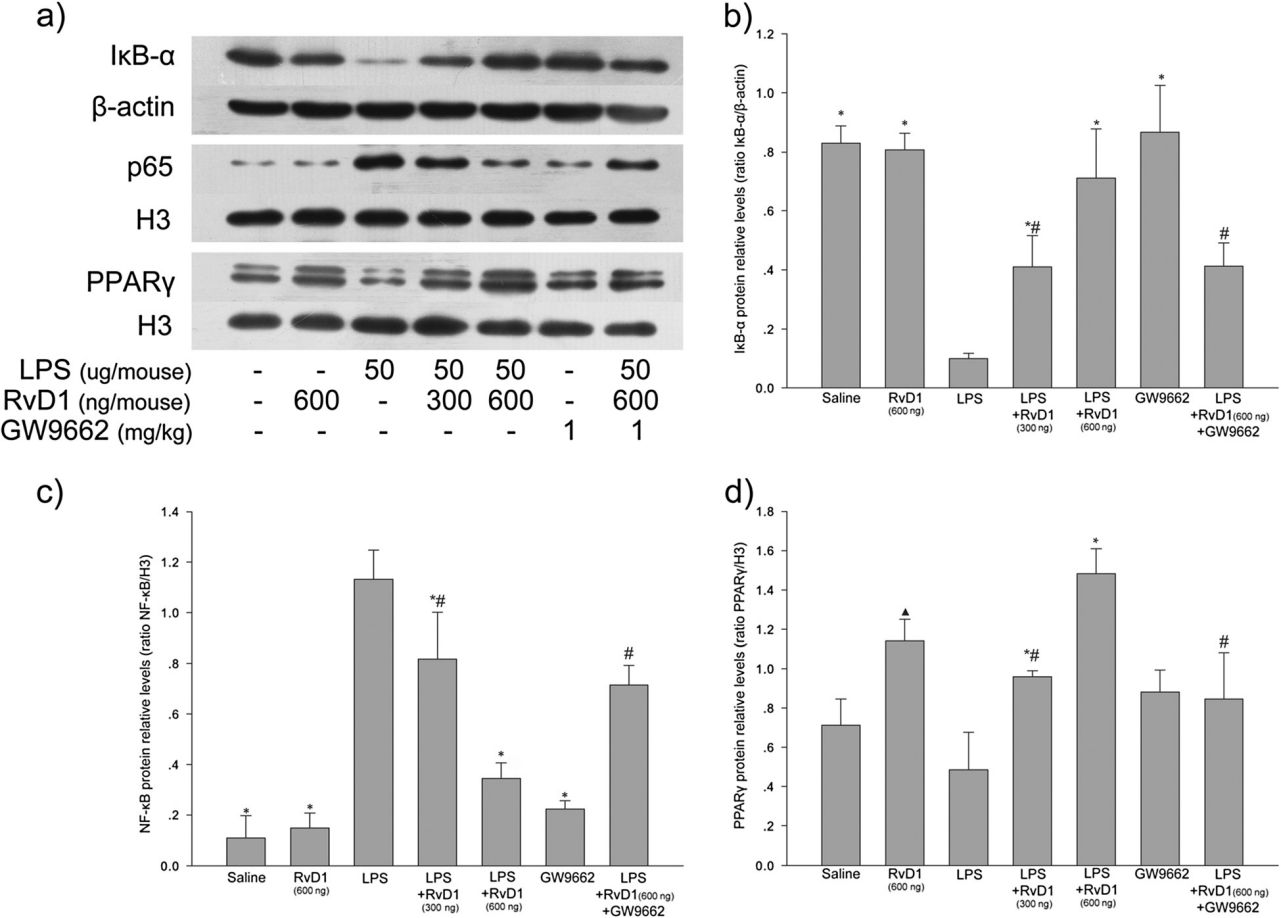
**Effect of RvD1 on LPS-induced changes in expression of IκBα, NF-κB p-65 subunit and PPARγ in lung tissues. (a)** Expression levels of IκBα, NF-κB p-65 and PPARγ. Histograms show mean ± S.D. (n = 3) of the relative intensity of **(b)** IκBα protein bands normalized to the β-actin band, **(c)** NF-κB p-65 protein bands normalized to the histone H3.1 band, and **(d)** PPARγ protein bands normalized to the histone H3.1 band. * p < 0.05 in comparison with the LPS group. # p < 0.05 in comparison with the LPS + RvD1 (600 ng) group. *black triangle* p < 0.05 in comparison with the saline group. Representative results of three independent experiments with different mice are shown.

Lung tissue samples were collected 6 h after LPS administration. LPS induced IκBα degradation and increased levels of NF-κB p65 subunit in the nucleus. RvD1 inhibited both of these effects of LPS in a dose-dependent manner (Figure 4, panels a and d). Administering GW9662 before injecting 600 ng RvD1 partially reversed RvD1-induced inhibition of NF-κB activation (Figure 4, panels a-c).

RvD1 also increased the levels of PPARγ in the nucleus, and this was partially reversed by pretreatment with GW9662 (Figure 4, panels a and d). These results suggest that inhibition of NF-κB activation by RvD1 is partially dependent on activation of PPARγ.

### RvD1 decreased LPS-induced DNA-binding activity of NF-ÎºB in lung tissue

**Figure 5:**
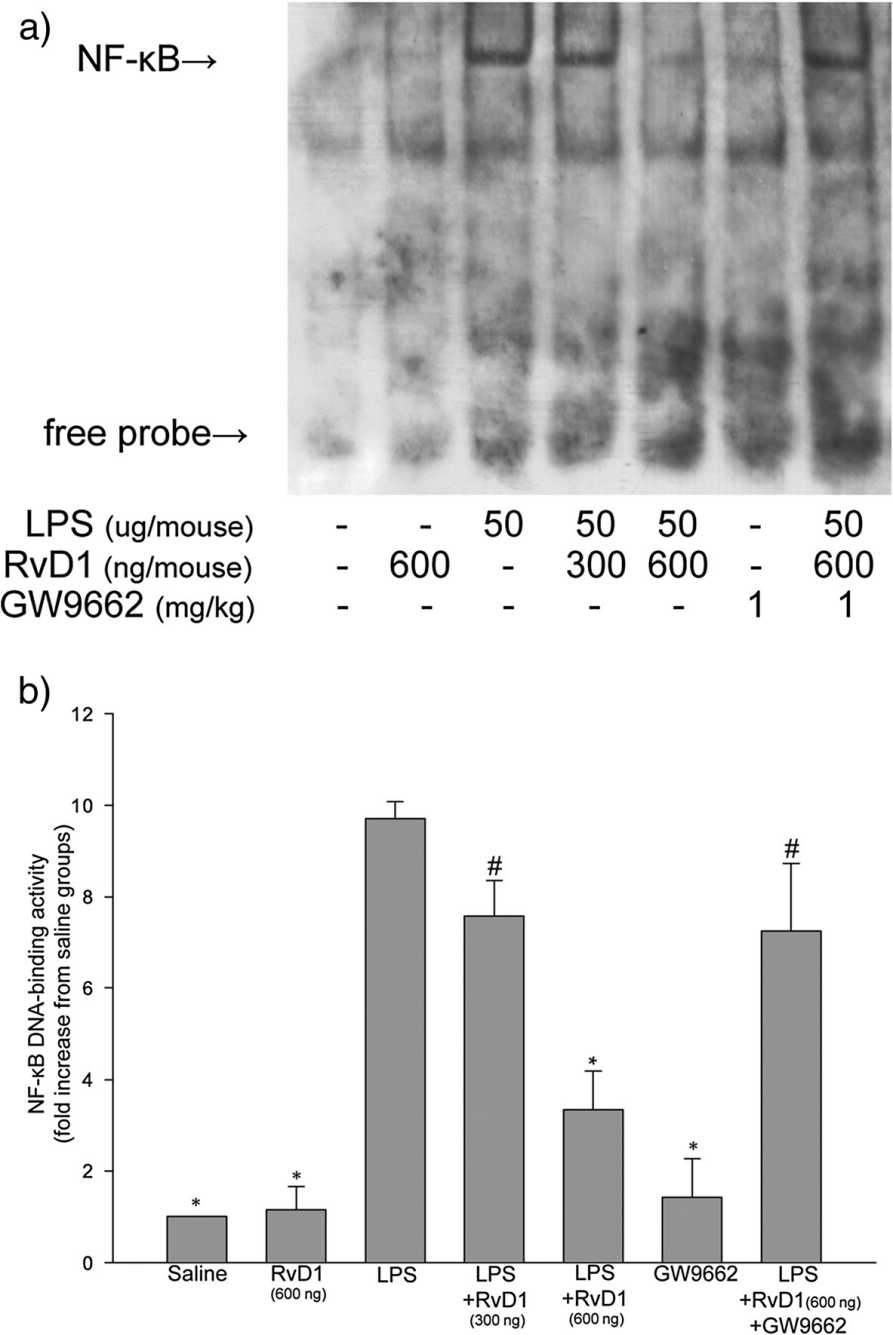
**Effect of RvD1 on the LPS**-**activated DNA**-**binding ability of NF**-**κB in lung tissue.** Lung tissue samples were collected at 6 h after LPS administration. **(a)** EMSAs were performed to detect the DNA-binding activity of NF-κB in nuclear extracts. **(b)** Histograms show the mean ± S.D. (n = 3) of the densitometric quantitation of DNA-binding activity by nuclear NF-κB relative to the control (saline). * p < 0.05 in comparison with the LPS group. # p < 0.05 in comparison with the LPS + RvD1 (600 ng) group. Representative results of three independent experiments with different mice are shown.

Lung tissue samples were collected at 6 h after LPS administration, and nuclear extracts were prepared. DNA-binding activity of NF-κB was relatively high in extracts from LPS-treated mice, whereas no binding was detected in extracts from saline-treated control mice (Figure 5). Administering 300 ng RvD1 led to a slight, nonsignificant decrease in NF-κB DNA-binding activity, while the higher dose of 600 ng led to significantly lower activity than in either the mice treated with LPS alone, or the mice treated with 300 ng RvD1 followed by LPS. Administering GW9662 before injecting 600 ng RvD1 partially reversed the RvD1-induced inhibition of NF-κB DNA-binding activity.

## Discussion

In our mouse model of LPS-induced ALI, pretreatment with RvD1 reduced levels of TNF-α, IL-6 and neutrophils in BALF, and it attenuated inflammation in lung tissues. RvD1 inhibited degradation of IκBα and it decreased the levels of NF-κB p65 subunit in the nucleus as well as the DNA-binding activity of nuclear NF-κB. Administering the PPARγ antagonist GW9662 before injecting RvD1 partially reversed these anti-inflammatory effects of RvD1. These results suggest that RvD1 may attenuate lung inflammation of LPS-induced ALI by suppressing NF-κB activation in a process that depends partly on PPARγ activation.

Proinflammatory cytokines play a critical role in ALI and ARDS: persistently elevated levels of proinflammatory cytokines such as TNF-α and IL-6 are associated with worse outcome in patients with ALI or sepsis [19]. Since TNF-α and IL-6 are considered markers of inflammation, the fact that their levels increased after LPS administration in our mouse model of ALI suggests that the model is valid.

Using this model, we found that RvD1 down-regulated levels of TNF-α and IL-6 in BALF of mice with LPS-induced ALI, and that it did so in a dose-dependent manner. These results are consistent with previous work in patients and animal models of disease. Feeding patients with rheumatoid arthritis or multiple sclerosis fish oils rich in ω-PUFA, from which RvD1 is derived, can reduce TNF-α and IL-6 levels [20]. RvD1 has been shown to reduce levels of TNF-α and IL-6 in mouse models of colitis, d-galactosamine-sensitized endotoxin shock and ALI [21-23]. In addition, the aspirin-triggered epimer of RvD1 (AT-RvD1; 7S,8R,17R-trihydroxy- 4Z,9E,11E,13Z,15E,19Z-docosahexzenoic acid) has been shown to decrease levels of TNF-α and IL-6 in a mouse model of hydrochloric acid-induced ALI [24].

Our findings, together with those of previous studies, suggest that RvD1 can down-regulate proinflammatory cytokines during the early stages of several inflammatory diseases, including ALI.

In many inflammatory diseases, neutrophils are rapidly recruited to sites of inflammation; there they kill invading bacteria through phagocytosis involving the release of preformed granular enzymes and proteins, as well as the production of a range of oxygen species. However, the highly destructive activity of neutrophils can also pose a risk to healthy tissues. This helps to explain why one of the first steps in the resolution of inflammation, which occurs when the initial injury or microbial invasion has been limited and injurious stimuli or microbes have been neutralized [25], is the loss of neutrophils from the inflamed area. In fact, research over the last decade has shown that resolution of inflammation is a programmed process that is actively regulated by various pro-resolving lipid mediators derived from ω-PUFA, including LXA4, RvE1, and PD1 [26]. In our mouse model of LPS-induced ALI, RvD1 at a dose of 600 ng/mouse significantly and rapidly reduced the numbers of neutrophils in BALF and attenuated inflammation and ALI scores in lung tissues.

RvD1 may decrease the numbers of neutrophils at inflammatory sites through the same mechanisms as other pro-resolving lipid mediators derived from ω-PUFA such as LXA4, RvE1, and PD1. Mechanisms proposed for these other mediators include: (i) limiting polymorphonuclear leukocyte (PMN) infiltration to inflamed sites, (ii) up-regulating CCR5 expression on apoptotic neutrophils, and (iii) enhancing apoptotic PMN engulfment by macrophages [26]. We speculate that RvD1 down-regulates neutrophils at inflammation sites through multiple mechanisms. One is that RvD1 exerts anti-inflammatory effects by down-regulating levels of TNF-α and IL-6, and perhaps also levels of neutrophil chemoattractants such as IL-8 and LTB4 [19]. In this way, RvD1 would reduce the number of neutrophils entering the BALF. At the same time, RvD1 exerts pro-resolving effects, as do other lipid mediators, by accelerating the exit of neutrophils from sites of inflammation. It may do this by up-regulating CCR5 expression on apoptotic neutrophils, stimulating their uptake by macrophages and the subsequent exit of phagocytes from the exudate via the lymphatic system [26]. This is of particular relevance to ALI, since ALI/ARDS involves extensive inflammation with PMN activation in lungs, accompanied by interstitial edema and an intense inflammatory response. Neutrophil apoptosis and clearance are delayed in sepsis and ARDS, which exacerbates inflammatory injury to the parenchyma [27]; as a result, enhancing neutrophil apoptosis in ALI can decrease mortality and ameliorate lung damage [28]. In sum, we suggest that the ability of RvD1 to reduce neutrophil numbers at inflammatory sites and to attenuate inflammation in lung tissue in our mouse model of ALI involves a combination of anti-inflammatory and pro-resolving mechanisms.

As a first step towards elucidating these mechanisms, we examined whether RvD1 down-regulates inflammatory mediators and neutrophils in our mouse model through a PPARγ/NF-κB pathway. PPARγ is a ligand-activated nuclear transcription factor involved in cellular differentiation, cancer, inflammation, insulin sensitization, atherosclerosis, and metabolic diseases, and it has been shown to inhibit NF-κB and to play important roles in several inflammatory processes [9]. Various PUFA, especially ω-PUFA, are natural ligands of PPARγ [29]. PPARγ is found in both the cytoplasm and nucleus under normal conditions; when activated, cytosolic PPARγ translocates to the nucleus, where it induces gene transcription [30,31]. In our study, RvD1 increased the level of PPARγ in the nucleus in a dose-dependent manner. Pretreatment with GW9662 partially reversed the RvD1-induced increase in nuclear PPARγ. A simple explanation of our results is that RvD1 activates cytosolic PPARγ, causing it to translocate into the nucleus, and that this activation occurs via a PPARγ-dependent pathway.

Using our animal model, we also found that RvD1 decreased the level of nuclear NF-κB p65 subunit and the DNA-binding activity of nuclear NF-κB. Pretreatment with GW9662 partially reversed the RvD1-induced inhibition of NF-κB activation. These results suggest that RvD1 inhibits NF-κB activation through a pathway at least partially dependent on PPARγ activation. These findings are consistent with several studies in animals and tissue culture. Oxidized eicosapentaenoic acid (EPA) and DHA, from which RvD1 is derived, have been shown to act as potent endogenous PPARγ ligands and to inhibit NF-κB DNA-binding activity in vitro and in vivo [32]. In fact, both EPA and DHA down-regulated LPS-induced activation of NF-κB via a PPARγ-dependent pathway in human kidney-2 cells [12]. Therefore, RvD1 may exert both its anti-inflammatory and pro-resolving effects through a PPARγ/NF-κB pathway. This is particularly relevant to ARDS, because inhibition of NF-κB activation in patients with the disease reduced not only the levels of proinflammatory mediators but also the numbers of activated resident neutrophils, thereby accelerating the resolution of lung injury [33].

Taken together, our results strongly suggest that RvD1 interacts with a PPARγ/NF-κB pathway, but whether that interaction occurs by direct binding of RvD1 to PPARγ remains unclear. Krishnamoorthy and coworkers [34] transiently transfected HEK-293 cells with expression vectors encoding PPARγ receptors coupled to Gal4 and found that RvD1 did not activate PPARγ directly. They also showed that RvD1 specifically interacted *in vitro* with both lipoxin A_4_ receptor ALX and orphan receptor GPR32. These results do not necessarily have to apply *in vivo*, so future studies should examine this question in animals.

Indeed future studies should clarify several limitations of the present work. First, our results do not identify the mechanism(s) by which RvD1 can reduce neutrophils in BALF. Second, inhibition of PPARγ only partially reversed the effects of RvD1, suggesting that other signaling pathways may help mediate the effects of the drug. For example, RvD1 has been found to protect mice from LPS-induced ALI by interacting with MAP kinases as well as with the NF-κB pathway [23]. Another study found that ω-PUFA exerted broad anti-inflammatory effects in monocytic RAW 264.7 cells and in primary intraperitoneal macrophages through stimulation of G protein-coupled receptor 120 (GPR 120) [35]. Future studies should consider these and other pathways when examining downstream effectors of RvD1 anti-inflammatory and pro-resolving activity.

## Conclusion

We demonstrate that RvD1 can inhibit NF-κB activation and attenuate lung inflammation in LPS induced-ALI. Inhibition of NF-κB activation by RvD1 was partially reversed by the PPARγ antagonist GW9662, which also reduced levels of PPARγ in the nucleus. These results suggest that RvD1 may attenuate lung inflammation in LPS-induced ALI by suppressing NF-κB activation via a pathway partially dependent on PPARγ activation. The powerful anti-inflammatory and pro-resolving effects of RvD1 observed here suggest that it has potential for clinical use.

## Abbreviations

ALI: Acute lung injury; ARDS: Acute respiratory distress syndrome; BALF: Bronchoalveolar lavage fluid; DHA: Docosahexaenoic acid; LPS: Lipopolysaccharide; PPARγ: Peroxisome proliferator-activated receptor gamma; RvD1: Resolvin D1; ω-PUFA: Omega-3 polyunsaturated fatty acids.

## Competing interests

Disclosure of conflicts of interest: We certify that all our affiliations with or financial involvement in, within the past 5 years and foreseeable future, any organization or entity with a financial interest in or financial conflict with the subject matter or materials discussed in the manuscript are completely disclosed.

## Authors’ contributions

ZL and JD contributed equally to designing the study, carrying out experiments, collecting and analyzing data, and writing the manuscript. These authors can vouch for the integrity of the data analysis. WW contributed to manuscript writing. TY carried out the Western blot analysis and contributed to data collection. TW contributed to data collection and manuscript writing. LG, LC and DX contributed to data collection. FW contributed to designing the study, analyzing the data, and writing the manuscript, and can vouch for the integrity of the data analysis. All authors read and approved the final manuscript.
